# Germination-Induced Variations in Proximate Composition, Phytochemical Profile and Antioxidant Activities of White Sesame Seeds: A Green Approach for Nutritional Modification

**DOI:** 10.3390/biotech15030060

**Published:** 2026-07-29

**Authors:** Oneeza Anwar, Iahtisham-Ul-Haq, Ramiz Arif, Waqas Ahmed, Hafiz Ansar Rasul Suleria

**Affiliations:** 1 Kauser Abdulla Malik School of Life Sciences, Forman Christian College (A Chartered University), Lahore 54600, Punjab, Pakistan; oneeza_anwar@outlook.com (O.A.); ramizarif09@gmail.com (R.A.); 2 Department of Food Science and Human Nutrition, University of Veterinary and Animal Sciences, Lahore 54000, Punjab, Pakistan; waqas.niaz@uvas.edu.pk; 3 School of Agriculture, Food and Ecosystem Sciences, Faculty of Science, University of Melbourne, Parkville, VIC 3010, Australia

**Keywords:** antioxidant activity, germination, phytochemical profile, sesame seeds

## Abstract

Germination is a cost-effective bioprocessing technique for enhancing seed properties such as sesame seeds, an underutilized seed crop. This research evaluates modifications in physicochemical and antioxidant activity of white sesame seeds (*Sesamum indicum* L.) influenced by germination. In this context, non-germinated seeds and germinated seeds at 24, 48, 72, 96, and 120 h were evaluated separately for their phytochemical profile, including TFC (aluminum chloride colorimetric method), TPC (Folin–Ciocalteu method) and antioxidant properties (DPPH and ABTS radical scavenging assays). Significant variations in the proximate composition, phytochemical densities, and antioxidant activities of white sesame seeds were observed as influenced by germination. The statistical analysis showed an increase in flavonoid content when germination was extended to 48 h, but a decline when extended beyond 48 h, whereas higher polyphenol quantities were observed in sesame seeds germinated for 96 and 120 h. The DPPH activity of germinated (24 h) sesame seed extracts was highest. In contrast, ABTS activity was higher in non-germinated seeds than in seeds germinated for 96 h, and it began to decline at 120 h. In general, a negative correlation between antioxidant activity and extended germination time (120 h) has been observed. For the incorporation of sesame seeds into functional food products, 48 h of germination appeared to have potential suitability for a favorable antioxidant and phytochemical profile.

## 1. Introduction

Germination is a regeneration process that converts a seed into a seedling [1]. The dried and dormant seed initiates the germination process by the absorption of water, and the process halts on the emergence of the radicle from the seed [2]. It is a traditional bioprocessing technique that reduces antinutritional compounds, improves the nutritional profile, thereby increasing consumer acceptability, and reduces the risk of allergic reactions [3,4].

With rising raw material costs, the pharmaceutical and nutraceutical industries have been paying greater attention to natural sources and novel crops for the treatment and improvement of human health. Sudden interest among researchers, nutritionists, and consumers has been observed in the use of germinated grains as a functional food [5]. Sesame seeds are an emerging crop providing numerous benefits due to their high levels of bioactive compounds [6].

Sesame (*Sesamum indicum* L.) is from the genus *Sesamum* and belongs to the family of *Pedaliaceae* [6]. Sesame seeds are a reliable substitute for animal protein because they contain both essential and nonessential amino acids. These contain 17–40% protein, compared with meat (18–25%) and cereals (7–13%), which is relatively high. Additionally, it is an excellent alternative to animal milk, containing essential mineral calcium. Sesame seeds have three times as much calcium as milk in the same amount. Sesame contains 15–18% hull, which is removed during processing; the remaining portion is the major source of protein and is added to animal feed.

Sesame seeds can act as an excellent source of nutrients to overcome micronutrient deficiencies [7]. These contain high levels of iron and calcium [8], and are an excellent source of bioactive compounds known as sesame lignans. The most dominant lignans in sesame seeds are sesamin, sesamolin, and sesaminol [9]. These have many therapeutic effects on human health; for example, consuming 60 mg of sesamin helps lower blood pressure [10]. Sesamin and sesamolin have neuroprotective, hypercholesterolemic, and anti-inflammatory effects [11]. Sesamin has anti-hypertensive properties, anti-obesity, and anti-thrombotic activities [10]. Sesamin is a bioactive compound in sesame seeds that helps protect the liver from oxidative damage. It also has antifungal, antiviral, antibacterial, and anti-inflammatory effects [12]. These have shown excellent anti-cancer, antioxidant, and cholesterol-improving properties [13]. Furthermore, these provide many technological and industrial uses and have extensive applications in the baking industry. Sesame seed hull and bran are used in animal feed. They have excellent emulsifying properties and oil- and water-holding capacity. Fortification with sesame seeds is done in bakery goods to improve the properties in the final product [14].

White sesame seeds are cultivated in warm-climate regions around the globe [15]. “This plant is an erect annual herb that has different names across various cultures, including *ajonjoli* (Spanish), *huma* (Chinese), *gergelim* (Portuguese), *goma* (Japanese), *sesame* (French), *til* (Hindi), and *konjed* (Persian)”, according to Nagar, et al. [16]. As stated by FAO, “estimated production of sesame seeds was 7,706,642 tons in 2020 which is 3.89% more than the previous year and 57.37% more than ‘10 years ago”. China is one of the major importers of these seeds. Being full of nutrients and still receiving no attention to improve its economic and nutritional value. Value addition should be considered for this underutilized rich oilseed [17]. Therefore, there is a growing need to explore new processing techniques, such as germination, to enhance the nutritional and economic value of white sesame seeds.

Germination activates enzymes (e.g., lipase, protease, and amylase), improves nutrient bioavailability, alters the grain’s morphology, and enhances the grain’s nutritional profile [18,19]. The enzymes break down proteins, lipids, and carbohydrates into their simpler forms [20]. Germination is divided into three stages: the first stage, known as imbibition, which involves absorption of water by the seed to hydrate it; the second stage involves the activation of metabolism; and the third stage is radicle protrusion, during which the radicle starts to appear because of the development of the seed embryo axis [21,22]. Germination produces new bioactive compounds, such as flavonoids and polyphenols, that further enhance antioxidant activity [23]. During germination, many metabolic changes take place. Starch is broken down into dextrin and maltose, which is then converted into glucose. The glucose obtained is transported to the hypocotyl to support germination and growth. Proteins are also degraded into free amino acids that are transported to the embryo, contributing to the formation of new cellular protoplasm. As germination progresses, starch content in grains declines, resulting in reduced peak [4]. Additionally, lipase is activated, leading to the breakdown of stored fats into fatty acids, and the glyoxylate cycle provides energy for radicle development [24].

The existing literature on sesame seed cultivars has been explored, but only for short durations (12–72 h). Extensive germination duration, like in this study, remains unreported. It studies the full span from imbibition to full radicle development before sprouts. At the later stages, there is a higher chance of fungal colonization under ambient conditions. This duration provides the full dataset window from start to end. White sesame seeds are predominantly consumed and commercially traded in developing countries. It is an economically accessible raw material with dense nutritional properties. Germination is therefore a critical pre-processing step that triggers enzymatic activation, reduces anti-nutritional factors, and restructures the seed’s biochemical composition. It is thought that without germination, the bioavailability of sesame seed bioactive compounds, including lignans, polyphenols, and flavonoids, remains limited, restricting their functional food potential. Optimizing germination duration is particularly important, as excessively prolonged germination may cause microbial contamination, color deterioration, and a decline in antioxidant capacity. Hence, the present study aimed to investigate the effect of varying germination durations (0 to 120 h) on the proximate composition, phytochemical profile, and antioxidant activities of white sesame seeds (*Sesamum indicum* L.), to identify the potentially suitable germination period for maximizing their nutritional and functional properties for incorporation into value-added food products.

## 2. Materials and Methods

### 2.1. Procurement of Raw Materials

White sesame (*Sesamum indicum* L.) seeds were procured from the local market in Lahore, Punjab, Pakistan. Sesame seeds were bought from a single vendor at one time and were visually screened to ensure uniformity across all seeds before buying. Sesame seeds were stored in airtight polythene bags at room temperature (25 ± 2 °C) until further use. The chemicals required for the analyses were purchased from Sigma-Aldrich (Taufkirchen, Germany) and Merck (Darmstadt, Germany).

### 2.2. Germination of Sesame Seeds

Sesame seeds were divided into six equal batches, each weighing 100 g. The sesame seeds were winnowed to remove any adhered hulls or damaged seeds from the sample. Sesame seeds were sorted manually to remove any stones or other extraneous particles. Subsequently, seeds were rinsed with water to remove the dust particles that had adhered. After washing them 2–3 times, the seeds were soaked in water for 5 h. The water was drained, and seeds were spread evenly across hard plastic trays (30 × 20 cm) lined with double-ply wet tissue paper for the germination process. Sesame seeds were sprayed with water twice a day and closely monitored to ensure no mold growth. The samples were kept for 120 h of germination at room temperature (25 ± 2 °C; relative humidity 30–35%). The germinated seeds were harvested every 24 h for 5 days. The seeds were dry-roasted in a pan over medium heat to halt enzymatic activity and the germination process. Non-germinated samples and germinated samples obtained at 24, 48, 72, 96, and 120 h were grouped for further testing, as shown in Table 1. The samples were kept in airtight bags at room temperature (25 ± 2 °C) until further evaluation.

### 2.3. Extraction of Phytochemicals

**Figure 1:**
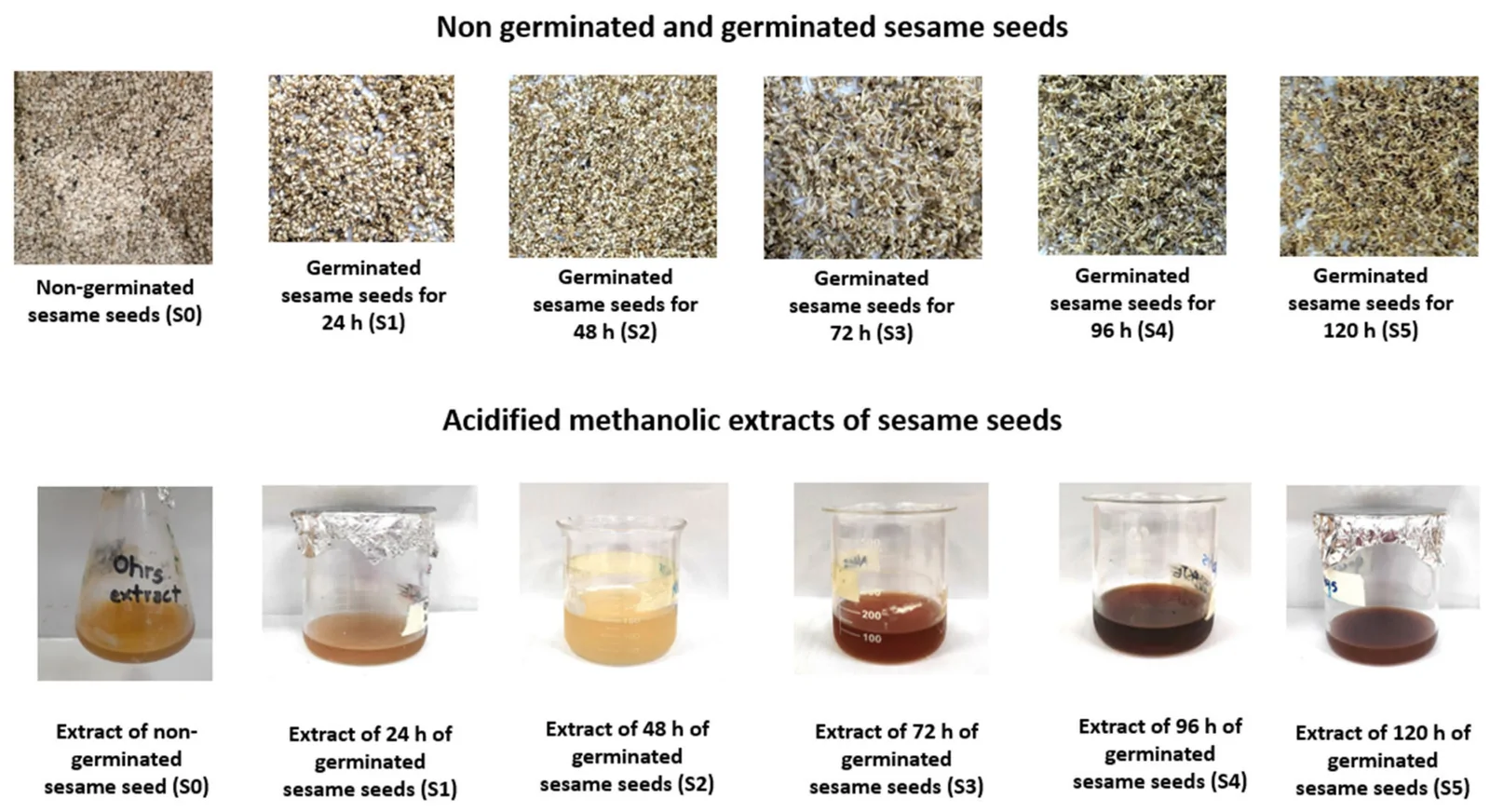
Non-germinated and germinated seeds of different durations.

The extracts of non-germinated and germinated sesame seed samples were prepared by drying the samples at 105 °C for 24 h until a constant weight was reached. The samples were processed into powder form using a pestle and mortar. The ground form was further utilized to make methanolic extracts. The acidified methanol solution was prepared using HCl (37%), double-distilled water, and methanol (99.9%) (1:10:80), respectively. Each extract consisted of a ground sample (15 g) dissolved in a solution of acidified methanol (150 mL). The dissolved samples were subjected to incubation at room temperature for 2.5 h and then incubated additionally for 2 h at 30 °C in a shaking incubator (Wise Cube WIS-30R, Daihan Scientific, Wonju-si, Gangwon-do, Korea) at 150 rpm. The incubated samples were filtered using double muslin cloth and then passed through a Whatman filter paper size 42 to ensure no sesame flour residue remained in the extract (Figure 1). The extracted samples were stored in reagent bottles to prevent methanol evaporation and refrigerated until further use. These extracts were used for the quantification of flavonoid content, phenolic content, and antioxidant activities.

### 2.4. Proximate Composition

#### 2.4.1. Dertermination of Moisture Content

To assess the total moisture content of sesame seeds, the AOAC [25] protocol (methods 92.12 and 41.1.02) was followed. Two grams of the weighed sample was placed in a pre-weighed Petri dish and kept in the forced-draft hot-air oven (SLN 115, POL-EKO, Poland) for 24 h at 105 °C until a constant weight was achieved.

Moisture percentage was calculated as per the expression below:

$$\mathrm{Moisture}\;\left(\%\right)\;=\;\frac{\mathrm{Weight}\;\mathrm{of}\;\mathrm{sample}\;\mathrm{before}\;\mathrm{drying}-\mathrm{Weight}\;\mathrm{of}\;\mathrm{sample}\;\mathrm{after}\;\mathrm{drying}}{\mathrm{Weight}\;\mathrm{of}\;\mathrm{sample}\;\mathrm{before}\;\mathrm{drying}}\;\times \;100$$

#### 2.4.2. Dertermination of Ash Content

The AOAC protocol [25] was followed to quantify the ash content (method 942.05, 4.1.10). Sesame seeds (2 g) were weighed before being added to the crucible. The weight of the crucible was also noted. The weighed sample was added to the crucible and charred over a high flame on a Bunsen burner until no fumes remained. The charred samples were put in a muffle furnace at 550 °C for 6 h. As the samples turned whitish gray, they were removed from the muffle furnace and kept in the desiccator until cooled. Ash content was determined by calculating the percentage of the sample weights before and after incineration.

$$\mathrm{Ash}\left(\%\right)=\frac{\mathrm{Weight}\;\mathrm{of}\;\mathrm{sample}\;\mathrm{after}\;\mathrm{incineration}}{\mathrm{Weight}\;\mathrm{of}\;\mathrm{sample}\;\mathrm{before}\;\mathrm{incineration}}\;\times \;100$$

#### 2.4.3. Dertermination of Crude Protein Content

The crude protein analysis was performed using the Kjeldahl method described by Goyal, et al. [26], with slight modifications. Digestion was performed by taking 2 g of the sample in the heating tube and mixing it with 20 mL of concentrated H_2_SO_4_ (98%). The sample was digested with 20 g of digestion mixture (K_2_SO_4_:FeSO_4_:CuSO_4_) in an 85:10:5 ratio, respectively. The sample was left to digest at 420 °C for 3 h, until it turned light green. Later, the volume of the digested sample was raised to 100 mL with distilled water. Afterwards, 10 mL of the diluted sample was distilled by treating it with a 4% boric acid solution, with methyl red as the indicator. Distillation was carried out for 7 min for each sample. After distillation, the sample was titrated against 0.1N HCl solution. The volume used for the back titration until the sample regained its original reddish color was used to determine the nitrogen percentage.

For the calculations, the nitrogen percentage was calculated by using the formula given below:

$$\mathrm{Nitrogen}\;(\%)\;=\frac{\mathrm{Vol}.\;\mathrm{of}\;0.1N\;\mathrm{HCl}\;\mathrm{used}\;\times \;0.0014\;\times \;\mathrm{Total}\;\mathrm{dilution}\;\mathrm{volume}}{\mathrm{Weight}\;\mathrm{of}\;\mathrm{sample}\;\times \;\mathrm{Volume}\;\mathrm{of}\;\mathrm{diluted}\;\mathrm{sample}\;\mathrm{taken}}\;\times \;100$$

Crude Protein (%) = Nitrogen (%) × 6.25

#### 2.4.4. Dertermination of Crude Fat Content

The fat content (method 948.22, 40.1.05) was evaluated by using the method explained by AOAC [25]. A sample (2 g) was weighed and placed into a thimble made of filter paper. The thimble was placed in the extraction chamber of the Soxhlet apparatus. The extraction process was carried out for 4 h. Hexane (95%) was used as a solvent. Later, the samples were removed and placed in the desiccator until a constant weight was reached and then further dried in a hot-air oven at 105 ± 2 °C until the final constant weight was achieved.

$$\mathrm{Crude}\;\mathrm{Fat}\;(\%)=\frac{\mathrm{Weight}\;\mathrm{of}\;\mathrm{the}\;\mathrm{sample}\;\mathrm{before}\;\mathrm{extraction}-\mathrm{Weight}\;\mathrm{of}\;\mathrm{the}\;\mathrm{sample}\;\mathrm{after}\;\mathrm{extraction}}{\mathrm{Weight}\;\mathrm{of}\;\mathrm{the}\;\mathrm{sample}\;\mathrm{before}\;\mathrm{extraction}}\;\times \;100$$

#### 2.4.5. Dertermination of Crude Fiber Content

The fiber was extracted by following the AOAC (962.09, 4.6) method [25]. Sesame seeds (2 g) were taken in already weighed glass crucibles. Once set, 150 mL of 1.25% H_2_SO_4_ (98%) was added to digest the protein. An amount of 3–5 drops of an antifoam agent (n-octanol) was added to ensure there was no foam. The sample mixture was heated at 100 °C for 30 min and then washed with deionized water. Subsequently, 150 mL of 1.25% NaOH was heated at 100 °C for 30 min. After washing it with 30 mL of hot deionized water, it was washed with cold deionized water to cool the sample and later washed with 25 mL of acetone (99.5%) thrice. The remaining samples in the crucibles were carefully removed. The samples were weighed into ceramic crucibles and left to dry for 3 h in the oven at 105 ± 2 °C. The fully dried samples were charred until no fumes remained and kept in the muffle furnace at 550 °C until a constant weight was reached. Once the sample turned gray, they were kept in a desiccator and weighed once cooled. The formula below was used to quantify the amount of fiber in percentage:

$$\mathrm{Crude}\;\mathrm{Fiber}\;(\%)\;=\frac{Weight\;of\;fiber\;content}{Initial\;weight\;of\;the\;sample}\;\times \;100$$

#### 2.4.6. Estimation of Nitrogen-Free Extract (NFE)

The subtraction method was used for nitrogen-free extract. All the values obtained from the proximate analysis were subtracted from 100.

The calculations were done using the following formula:

NFE (%)=100−(moisture content + ash content + crude protein + crude fat + crude fiber)

### 2.5. Phytochemicals

#### 2.5.1. Total Phenolic Content (TPC)

To determine the total phenolic content in the sesame seed extracts, the procedure devised by [27] was followed. The extracts (0.1 mL) were added to the test tubes. Afterward, double-distilled water (6 mL) was added. Then, 0.5 mL of Folin–Ciocalteu Reagent (2M) and 20% sodium carbonate solution (1.5 mL) were added to the extracts. Lastly, double-distilled water (1.9 mL) was added to the tubes and mixed to ensure a homogeneous mixture. The homogenized reaction mixture was incubated for 30 min at 40 °C, and the spectrophotometer (Shimadzu UV-1900I, Kyoto, Japan) was used to measure the absorbance of samples at 765 nm (Table S1). For the standard, gallic acid was used; different concentrations were prepared from a 1000 ppm stock solution, i.e., 0–100 µg/mL. Acidified methanol served as a blank. The coefficient of determination (R^2^) used to calculate TPC is given below:

Y = 0.0095x − 0.0315;  R^2^ = 0.9731;

LOD = 11.93 µg/mL; LOQ = 36.17 µg/mL

Repeatability = 1 to 4.71%; Pooled Precision = 2.21%

The final results were expressed as mg GAE/100g of sesame seeds.

#### 2.5.2. Determination of Total Flavonoid Content (TFC)

The procedure reported by Heimler, et al. [28] was used to determine the total flavonoid content. The sesame extracts (250 μL) were diluted by adding 1.25 mL of double-distilled water to the test tubes. A 5% sodium nitrite solution (75 μL) was added to each diluted extract. The diluted extracts were incubated for 6 min, and then a 10% AlCl_3_ solution (150 μL) was added to each test tube. This mixture was incubated for 5 min, then 0.5 mL of 1 M NaOH was added. For the quantification of total flavonoid content, quercetin was used as a standard, and different standard concentrations were made from the 1000 ppm stock solution, i.e., 0–400 µg/mL. Absorbance was recorded at 510 nm for the samples (Table S1) by a spectrophotometer (Shimadzu UV-1900I, Kyoto, Japan). The coefficients of determination used for calculations of TFC are given below:

Y = 0.0008x + 0.0477;   R^2^ = 0.9991

LOD = 11.38 µg/mL;  LOQ = 34.51 µg/mL

Repeatability = 1.01 to 4.69%; Pooled Precision = 2.38%

The final results were expressed as mg QE/100g of sesame seeds.

To estimate the individual flavonoid content in sample extracts, the given formula was used. The results of individual flavonoids were represented as UV-vis estimates.

$$\mathrm{Flavonoid}\;(\mathrm{mg}/L)=\frac{A\times \;\mathrm{DF}\;\times \;\mathrm{MW}\;\times \;1000}{\varepsilon \;\times \;l}$$

A = absorbance taken at specific wavelength; DF = dilution factor; MW = molecular weight of the specific flavonoid; ε = extinction coefficient; l = path length.

Respective wavelengths, extinction coefficients, and molecular weights of prominent flavonoids are enclosed in Table 2.

### 2.6. Antioxidant Assay Test

#### 2.6.1. Determination of DPPH Radical Scavenging Activity

The antioxidant activity of the germinated and non-germinated sesame seeds was evaluated by using 2,2-diphenyl-1-picrylhydrazyl (DPPH) radical scavenging ability by following the protocol adopted by Vulić, et al. [33]. For this purpose, 1 mL of sesame extract, 3 mL of methanol (99.9%), and 1 mL of freshly prepared 1 mM DPPH solution were mixed together. The prepared mixture was homogenized with a vortex mixer for 1 min and then left for 10 min at 27 °C. The absorbance was analyzed by a spectrophotometer (Shimadzu UV-1900I, Kyoto, Japan) at 517 nm (Table S2). Methanol was used as the blank, namely the control. The free radical scavenging activity of DPPH was expressed as a percentage as

$$\mathrm{DPPH}\;\mathrm{radical}\;\mathrm{scavenging}\;\mathrm{activity}\;(\%)=\frac{A-B}{A}\times 100$$

A defines the absorption value of the control reaction mixture, whereas B defines the absorption value of the sample reaction mixture.

#### 2.6.2. Determination of ABTS^+^ Free Radical Scavenging Activity

The protocol stated by Raupp, et al. [34] was followed to quantify the antioxidant activity of sesame seed extracts. Equal proportions of 7 mM aqueous ABTS and 2.45 mM Potassium persulfate were mixed to make the solution. K_2_S_2_O_8_ was used for the activation of ABTS and left to incubate for 16 h in the dark at 4 °C. Before analysis, the ABTS solution was diluted with methanol (99.9%) to obtain an absorbance between 0.8 and 1 at 734 nm. Then, the methanolic extract (20 μL) was mixed with the ABTS reagent (3 mL) and incubated in a water bath at 30 °C for 30 min. After incubation, the color of the reaction mixture faded. Finally, a spectrophotometer (Shimadzu UV-1900I, Kyoto, Japan) was used to measure absorbance at 734 nm (Table S2). The final results were reported as percent inhibition of ABTS radical by comparing the absorbances with its control.

### 2.7. Color Analysis

The color analysis of the sesame seeds was recorded by using a hand-held colorimeter (Konica Minolta, CR-10, Tokyo, Japan). The CIE lab system was used to evaluate L, a*, and b* values (Table S3). Furthermore, chroma and hue angle were calculated. The analysis was done at room temperature and under white fluorescent light.

$$C\mathrm{hroma}=\sqrt{{\left(a^{*}\right)}^{2}+{\left(b^{*}\right)}^{2}}$$

$$\mathrm{Hue}\;\mathrm{angle}=\tan^{-1}\left(\frac{b^{*}}{a^{*}}\right)$$

### 2.8. Statistical Analysis

All the data were analyzed for statistical significance using analysis of variance (ANOVA) for continuous quantitative data. Further, the means for each parameter were compared using Tukey’s Honest Significant Difference (HSD) test. Statistix 8.1 software was used to analyze the data. MS Excel was used for Pearson’s Correlation Analysis. GraphPad Prism (version 8) was used to generate heatmaps. The level of significance was set at *p* < 0.05. All analyses were performed in triplicate, and the values were expressed as mean ± standard deviation.

## 3. Results

### 3.1. Proximate Analysis of Sesame Seeds

The statistical results for the proximate composition of non-germinated and germinated sesame seeds are represented in Table 3. The details about their results are discussed below.

#### 3.1.1. Moisture Content

The moisture content showed significant differences (*p* < 0.01) among the treatments. It showed varied results, as the maximum moisture content observed was at S3 (sesame seeds germinated for 72 h) as 10.05 ± 0.15%, followed by S2 (sesame seeds germinated for 48 h) as 9.88 ± 0.22%. S0 (non-germinated sesame seeds) showed the minimum amount of moisture, i.e., 4.39 ± 0.09%. A gradual increase in moisture content was observed in S1 (sesame seeds germinated for 24 h), followed by S4 (sesame seeds germinated for 96 h) and S5 (sesame seeds germinated for 120 h), with the moisture content as 6.59 ± 0.22%, 9.68 ± 0.16%, 9.63 ± 0.24%, respectively. The varied moisture content results may be due to substantial differences in water-binding properties or processing techniques among the treatments.

#### 3.1.2. Ash Content

The statistical evaluation of ash content indicated significant differences among the treatments (*p* < 0.01). It ranged from 1.32% to 2.12%. The minimum ash content noted was in sesame seeds germinated for 72 h (S3), and the maximum content was observed in sesame seeds germinated for 24 h (S1), indicating a greater mineral content than other treatments. S2 (sesame seeds germinated for 48 h) showed a decline in ash content (1.74 ± 0.19%). At S4, the ash content showed an increase after declining at S3 (1.81 ± 0.29%). The ash content of S5 and S5 is documented as 1.49 ± 0.01%, and S4 and S5 indicated they have intermediate ash content compared with other treatments. S2 and S4 were not significantly different.

#### 3.1.3. Crude Protein Content

The crude protein content showed highly significant (*p* < 0.01) results. Non-germinated seeds (S0) had the lowest protein content, at 18.64 ± 0.37%, increasing slightly to 19.57 ± 0.65% after 24 h of germination (S1), though S0 and S1 are statistically non-significant. An increase appeared in sesame seeds germinated for 48 h (S2), reaching 20.99 ± 0.46%, followed by further increase at 72 h and 96 h (S3 and S4), with values of 21.81 ± 0.33% and 22.00 ± 0.37%, respectively. The highest protein content was recorded at 120 h (S5), at 22.55 ± 0.56%. This gradual rise in crude protein throughout germination is largely attributed to the synthesis of new enzymes and proteins needed to support metabolic activity and seedling growth.

#### 3.1.4. Crude Fat Content

The crude fat content showed significant differences (*p* < 0.01) among the treatments. Non-germinated seeds (S0) had the highest fat content, i.e., 45.33 ± 1.76%, and this declined to 37.33 ± 1.26% after 24 h of germination (S1). The decline continued through 48 h and 72 h (S2 and S3), with values of 30.50 ± 1.00% and 26.67 ± 1.61%. At 96 h (S4), fat content declined further to 22.33 ± 1.53%, reaching its lowest point at 120 h (S5), at 19.17 ± 1.04%; S4 and S5 were statistically non-significant. This decrease in crude fat throughout germination reflects the breakdown of stored lipid reserves, as the seed grows on these reserves to meet embryo growth requirements.

#### 3.1.5. Crude Fiber Content

The treatments showed statistically significant (*p* < 0.01) differences in crude fiber content. Non-germinated seeds (S0) had the lowest crude fiber, i.e., 5.91 ± 0.12%, and it increased sharply, reaching 10.90 ± 0.36% at S1 (sesame seeds germinated for 24 h). The increase in fiber content continued through 48 h and 72 h of germination (S2 and S3), with values of 12.38 ± 0.27% and 12.63 ± 0.19%. At 96 h (S4), fiber content decreased slightly to 11.49 ± 0.20%, and a minor increase was observed at S5 (12.53 ± 0.31%). This general increase in fiber content is likely due to the enzymatic breakdown of stored reserves and growth of structural cell wall material as germination duration increases.

#### 3.1.6. Nitrogen-Free Extract (NFE)

Nitrogen-Free Extract (NFE) depicted highly significant (*p* < 0.01) results among all treatments. The lowest value appeared at 24 h (S1), at 23.49 ± 1.52%, while the highest was reached after 120 h (S5), at 34.64 ± 2.01%. From S0 through S3, NFE stayed steady, hovering between 23.49 ± 1.52% and 27.53 ± 2.42%, with no meaningful separation between these treatments. A clear jump occurred between S3 and S4, where NFE rose to 32.69 ± 1.13%, holding steady into S5 at 34.64 ± 2.01%; these last two values were statistically alike but clearly higher than the rest. Since NFE is derived by difference rather than measured directly, this rise mirrors the corresponding decline in other proximate components later in germination.

### 3.2. Phytochemical Profiling

The statistical results for the phytochemical profile of non-germinated and germinated sesame seeds are represented in Table 4.

#### 3.2.1. Total Polyphenol Content (TPC)

The treatments showed highly significant (*p* < 0.01) differences in total polyphenol content (TPC). The TPC in S0 (non-germinated sesame seeds) was 29.93 ± 0.73 mg GAE/100 g DW. TPC observed in S1 (germinated sesame seeds for 24 h) was 33.99 ± 3.32 mg GAE/100 g DW. The values from S0 to S2 are statistically non-significant. The mean for 48 h (S2) is 26.18 ± 3.6 mg GAE/100 g DW. Germination for 72 h (S3) depicted an increase in TPC to 58.42 ± 1.97mg GAE/100 g DW. S4 germinated for 96 h showed the value 90.42 ± 6.61 mg GAE/100 g DW, and S5 germinated for 120 h showed the highest TPC (137.28 ± 6.01 mg GAE/100 g DW) among all the treatments. A direct relation between germination time and polyphenol concentration; longer germination time significantly increases the polyphenols in the sesame seeds.

#### 3.2.2. Total Flavonoid Content (TFC)

The TFC of white sesame seeds varied significantly across all treatments, ranging from 11.94 to 53.15%. Non-germinated sesame seeds showed a TFC content of 46.66 ± 3.37 mg QE/100 g DW. Later, sesame seeds were germinated for 48 h (S2), showing that a shorter germination duration progressively increases TFC (53.15 ± 3.37 mg QE/100 g DW). The increase after germination may be due to the activation of the phenylpropanoid pathway. Later, TFC declined sharply across all treatments. The TFC of S1, S3, S4, and S5 are as follows: 28.5 ± 5.71 mg QE/100 g DW, 37.08 ± 0.78 mg QE/100 g DW, 21.04 ± 3.29 mg QE/100 g DW, and 11.94 ± 3.91 mg QE/100 g DW. The decline after germination suggests that higher germination duration reduces TFC content in sesame seeds.

#### 3.2.3. Flavonoid Profile

The statistical results for the UV-Vis-based estimation of the flavonoid profile of non-germinated and germinated sesame seeds are represented in Table 5. Rutin content showed a non-linear result across all treatments. They exhibited significantly different results (*p* < 0.01). Non-germinated seeds had a rutin content of 0.070 ± 0.006 mg/100 g DW. Later, this declined at S1 (germinated for 24 h), i.e., 0.058 ± 0.004 mg/100 g DW; the highest peak was noted at S2 (germinated for 48 h), 0.110 ± 0.008 mg/100 g DW; and the peak at 48 h can be associated with flavonoid glycosylation enzymes. Beyond S2, rutin content declined, as S3 showed rutin content as 0.094 ± 0.002 mg/100 g DW, S4 as 0.049 ± 0.002 mg/100 g DW, and S5 as 0.056 ± 0.002 mg/100 g DW. Longer durations are not favorable for rutin content.

The isorhamnetin content showed a significant (*p* < 0.01) difference among the treatments. S0 (non-germinated sesame seed) and S1 (sesame seeds germinated for 24 h showed no significant difference in isorhamnetin content, at 6.95 ± 0.52 mg/100 g DW and 6.23 ± 0.57 mg/100 g DW, respectively. The content of isorhamnetin started to increase after 24 h of germination (S2), i.e., 7.40 ± 0.39 mg/100 g DW. Isorhamnetin content was highest at S3, at 8.91 ± 0.32, suggesting mid-germination yields the highest isorhamnetin content, as it declined progressively in later stages. Beyond S3, it declined significantly, reaching 4.30 ± 0.27 mg/100 g DW in S4 (sesame seeds germinated for 96 h) and 4.93 ± 0.46 mg/100 g DW at S5 (sesame seeds germinated for 120 h).

Luteolin levels varied significantly (*p* < 0.01) across different germination stages of sesame seeds. S0 and S1 showed non-significant differences in luteolin content, with values of 1.50 ± 0.11 mg/100 g DW and 1.34 ± 0.13 mg/100 g DW, respectively. A prominent increase was observed in S2 (sesame seeds germinated for 48 h), i.e., 2.23 ± 0.08 mg/100 g DW, representing a 48.7% increase compared to S0. This change may be attributed to the phenylpropanoid pathway during mid-germination. After S2, a progressive decline was observed in S3, S4, and S5, at 1.92 ± 0.07 mg/100 g DW, 0.93 ± 0.06 mg/100 g DW, and 1.06 ± 0.10 mg/100 g DW, respectively. Suggesting later stages of germination reduced luteolin content in white sesame seeds.

The kaempferol content differed significantly (*p* < 0.01) among the treatments. It showed a non-linear trend among the treatments, as S0 (non-germinated sesame seeds) reported a kaempferol content of 0.96 ± 0.05 mg/100 g DW, which later decreased in sesame seeds germinated for 24 h (S1). S0 and S1 were non-significant. An increase in S2 (sesame seeds germinated for 24 h) was reported as 1.43 ± 0.14 mg/100 g DW. This increase is most probably due to the activation of flavanol synthase. At S3 (sesame seeds germinated for 72 h), the recorded kaempferol content was 1.20 ± 0.06 mg/100 g DW, and it further decreased to 0.66 ± 0.05 mg/100 g DW in S4, to 0.58 ± 0.02 mg/100 g DW in S5.

Quercetin increased during sesame seed germination with significant (*p* < 0.01) results. Quercetin was the most abundant individual flavonoid among all. It ranged from 58.21 to 3.57%. S0 showed quercetin content as 94.2 ± 7.12 mg/100 g DW, which slightly decreased in S1 to 84.45 ± 7.77 mg/100 g DW. A significant increase was observed in S2 to 140.04 ± 5.24 mg/100 g DW. After S2, a linear decline was observed from 120.74 ± 4.3 mg/100 g DW in S3 to 58.21 ± 3.57 mg/100 g DW in S4 and 66.78 ± 6.20 mg/100 g DW in S5, respectively. The peak at S3 is also due to the activation of flavanol synthase that mainly occurs during mid-germination.

The procyanidin content showed a notable difference (*p* < 0.01) among the treatments. Like the above results, S0 and S1 showed non-significant results. A significant increase was observed at S2 to 21.64 ± 0.70 mg/100 g DW, the highest procyanidin content among all treatments. At S3, the content declined to 19.14 ± 0.78 mg/100 g DW. The observed content of S4 and S5 decreased further to 9.64 ± 0.48 mg/100 g DW and 3.11 ± 0.10 mg/100 g DW, respectively.

β-Catechin showed a highly significant (*p* < 0.01) difference among the treatments. S0 and S1 remained statistically non-significant, with contents of 13.19 ± 0.59 mg/100 g DW and 12.42 ± 0.94 mg/100 g DW, respectively. The β-catechin content rose progressively at S2 to 19.97 ± 1.65 mg/100 g DW, representing a 51.4% increase compared with non-germinated sesame seeds. It acts as a precursor in the condensed tannins and procyanidin pathway. At S3, the β-catechin content continued to decline until S5, reaching 17.31 ± 0.83 mg/100 g DW, 8.01 ± 0.43 mg/100 g DW, and 9.07 ± 0.45 mg/100 g DW, respectively.

Epicatechin content varied significantly (*p* < 0.01) across different germination stages of sesame seeds. Non-significant results were obtained for S0 and S1, at 13.87 ± 1.19 mg/100 g DW and 11.55 ± 0.64 mg/100 g DW, respectively. Like all other individual flavonoids, epicatechin peaked at S2 as 21.74 ± 1.63 mg/100 g DW. Epicatechin content started decreasing at S3, reaching 18.42 ± 0.48 mg/100 g DW. A drastic decline was observed in S4 and S5 to 1.64 ± 0.04 mg/100 g DW and 1.13 ± 0.02 mg/100 g DW, respectively.

### 3.3. Antioxidants Assay Test

#### 3.3.1. DPPH Antioxidant Activity

The DPPH activity showed a significant variation (*p* < 0.01) among the treatments. A notable difference was observed between germinated and non-germinated sesame seeds. S0 (non-germinated sesame seeds) showed a DPPH percentage of 78.58 ± 0.16%. A sudden increase in the DPPH percentage was observed after 24 h of sesame seed germination (S1): 91.36 ± 0.24%. After S1, a linear decline was observed from S2 (sesame seed germinated for 48 h) until S5 (sesame seed germinated for 120 h) (82.07 ± 1.01%, 77.78 ± 1.20%, 30.22 ± 0.74%, 11.26 ± 0.29%), and S5 was the lowest among all (Table 6). The above percentages indicate that extended germination can reduce DPPH activity. The sudden decline observed at S4 and S5 can be attributed to oxidative degradation of antioxidants and polyphenols, as well as possible microbial consumption of antioxidants.

#### 3.3.2. ABTS^+^ Free Radical Scavenging Activity

**Figure 2:**
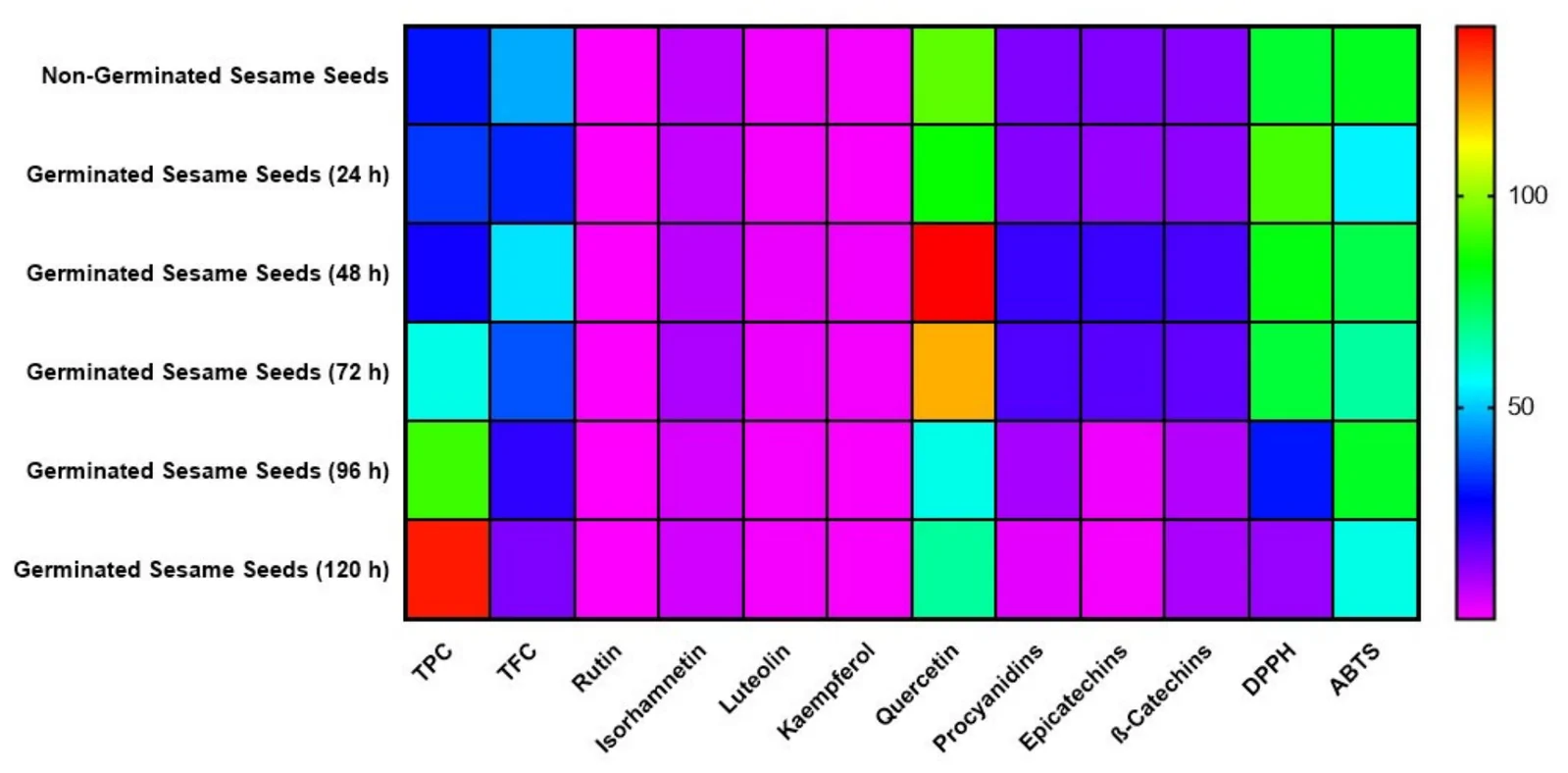
Heatmap for phytochemical and antioxidant profile of germinated and non-germinated sesame seeds.

The ABTS Activity showed a highly significant result (*p* < 0.01) across all treatments. Non-germinated sesame seeds (S0) had a percentage of 80.68 ± 1.81%. S1 (sesame germinated for 24 h) showed a similar percentage (54.89 ± 0.99%). An increase till 48 h of germination of sesame seeds (S2). A decline was observed in S3 (sesame seed germinated for 72 h), and later S4 (sesame seed germinated for 96 h) showed an increase from 66.51 ± 1.80% to 79.9 ± 0.16%. Sesame seeds germinated for 120 h (S5) depicted a decrease in ABTS percentage (58.74 ± 2.15%). Through the above results, S0 showed the highest ABTS percentage, followed by S4. The heatmap shows the relative densities of the phytochemical and antioxidant capacities of germinated and non-germinated sesame seeds (Figure 2).

#### 3.3.3. Correlation Analysis for Phytochemicals and Antioxidant Activities

**Figure 3:**
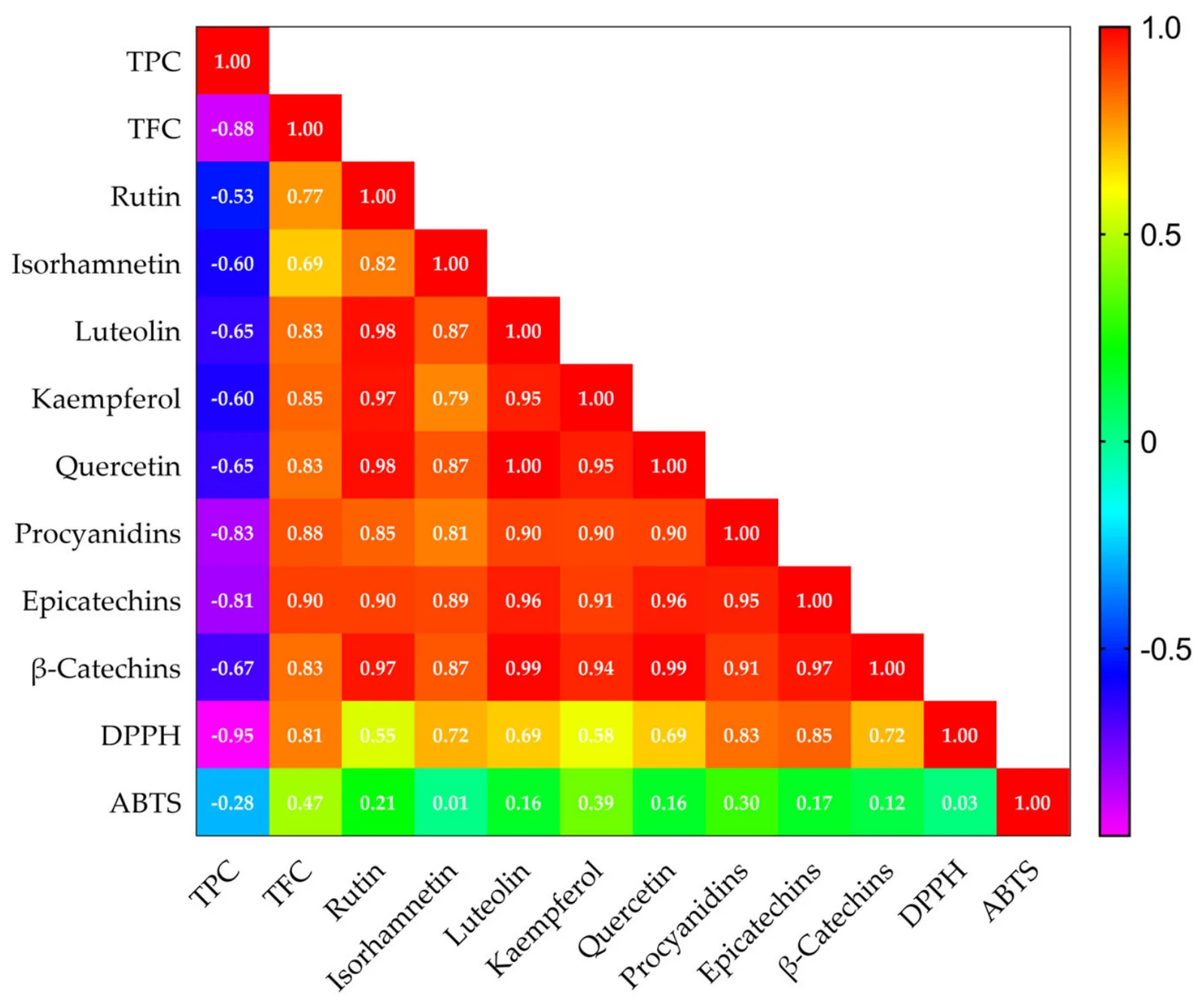
Correlation matrix for phytochemical and antioxidant profile of germinated and non-germinated sesame seeds.

The Pearson correlation matrix (Figure 3) reveals specific relationships among phenolic compounds and their associated antioxidant activities, providing insight into the biochemical interaction resulting in the radical scavenging potential of sesame seeds. Total polyphenols exhibit strong negative correlations with most flavonoid contents (r = −0.88 with total flavonoids; −0.95 with DPPH), suggesting that samples richer in polyphenols may contain a distinct composition of non-flavonoid phenolics that contribute differently to antioxidant capacity. On the other hand, total flavonoids show positive associations with individual flavonoid compounds such as rutin (r = 0.77), luteolin (r = 0.83), kaempferol (r = 0.85), and quercetin (r = 0.83–0.99), indicating that these molecules coexist and collectively contribute to the flavonoid fractions. The very strong correlations among luteolin, quercetin, and kaempferol (r > 0.95) imply structural or biosynthetic linkage, as these flavones and flavonols often share common metabolic pathways. Procyanidins and catechins (epicatechin and β-catechin) also correlate strongly with total flavonoids (r = 0.88–0.90) and with each other (r > 0.94), reflecting their polymeric nature and mutual contribution to antioxidant potential. These compounds are known to enhance radical scavenging through hydrogen donation and metal chelation, mechanisms that align with their high correlation to DPPH activity (r = 0.83–0.85). DPPH radical scavenging shows the strongest inverse relationship with total polyphenols (r = −0.95), suggesting that higher polyphenol concentrations correspond to greater antioxidant capacity, possibly due to synergistic effects among phenolic acids and flavonoids. ABTS activity, however, displays weaker correlations (r < 0.47 with most compounds), implying that the ABTS assay may respond differently to hydrophilic versus lipophilic antioxidants. Collectively, the data indicate that flavonoids, particularly quercetin, luteolin, and catechins, are key contributors to antioxidant performance, while total polyphenols represent a broader pool of compounds influencing radical scavenging efficiency through complementary mechanisms.

### 3.4. Color Profile of Germinated and Non-Germinated Sesame Seeds

#### 3.4.1. L

Statistical evaluation indicated that the treatments differed significantly (*p* < 0.01) in L value. The highest L value reported was for sesame seeds germinated for 24 h (S1), at 69.77 ± 1.17, followed by non-germinated seeds (S0) at 68.80 ± 0.10; both samples were light in color. The color began to decrease in other germinated samples, indicating darkening. The values showed a decreasing trend from S1 to S5. The value of S1 was 69.77 ± 1.17, followed by sesame seeds germinated for 48 h (S2) as 65.77 ± 1.18, S3 (germinated for 72 h) as 64.57 ± 2.83, S4 (germinated for 96 h) as 59.57 ± 4.16, and S5 (germinated for 120 h) as 59.95 ± 1.63 (Table 7). The decreasing trend shows that longer germination affects the color profile of the sesame seeds.

#### 3.4.2. a*

a* values showed significant (*p* < 0.01) differences among all treatments. The highest a* value obtained was for S1 (germinated sesame seeds for 24 h), at 4.35 ± 0.15, indicating a reddish color tone. The lowest a* value reported was for S3 (sesame seeds germinated for 72 h), at 1.97 ± 0.15, indicating a lightness in the red tone. Sesame seeds germinated for 48 h (S2) were 2.20 ± 0.20, whereas a* for sesame seeds germinated for 96 h (S4) and 120 h (S5) increased significantly (2.05 ± 0.05 and 2.30 ± 0.40). Elongated germination reduces a* values.

#### 3.4.3. b*

b* values showed highly significant (*p* < 0.01) variation among all treatments. The b* value of S0 (non-germinated sesame seeds) showed a yellowish tone (21.47 ± 0.67). The samples from S0 to S2 (germinated sesame seeds for 48 h) showed an increase. Sesame seeds germinated for 96 h (S4) and 120 h (S5) showed a decreasing trend, with values of 21.93 ± 1.77 and 19.37 ± 1.14, respectively. Longer germination makes the sample color darker.

#### 3.4.4. Chroma

All treatments showed highly significant differences in chroma (*p* < 0.01). Non-germinated sesame seeds (S0) showed a chroma value of 50.73 ± 0.81. S1 (sesame seeds germinated for 24 h) and S2 (sesame seeds germinated for 48 h) showed values of 54.9 ± 2.75 and 56.8 ± 0.80. A decline in chroma value was observed in sesame seeds germinated for 72 h (S3) to 120 h (S5), with values of 48.67 ± 1.86, 47.97 ± 3.54, and 43.33 ± 1.51. A longer germination period is causing the color intensity to decline.

#### 3.4.5. Hue Angle

Hue angle showed statistically significant (*p* < 0.01) results. The hue value of non-germinated sesame seed (S0) was 5.55 ± 0.71. A slight decline was observed in sesame seeds germinated for 24 h (S1), with the hue value of 5.32 ± 0.45. A notable increase (11.98 ± 1.12) in sesame seeds germinated for 48 h (S2) was observed. Sesame seeds germinated for 72 h (S3), 96 h (S4), and 120 h (S5), followed by a declining trend, i.e., 11.40 ± 0.50, 10.70 ± 0.88, and 8.65 ± 1.99. Longer germination directly affected the hue angle, causing the samples to darken.

## 4. Discussion

Germination increases moisture content; a similar trend was observed in the study by Mares, et al. [35], which explored the relation of germination time with chemical and antioxidant activity of white sesame seeds. Moisture was directly linked to longer germination duration. The observed increase in moisture in this study can be attributed to germination, which causes water absorption by the seeds [35]. The ash content increased significantly in the current study, which may be due to the conversion of carbohydrates into carbon dioxide during respiration [36]. The reported increase in ash content can be because of the formation of rootlets during germination [37]. The decrease observed during germination of sesame seeds can be attributed to mineral loss or increased water absorption by the germinated seeds, which dilutes the overall ash content [35].

The protein content in this study demonstrated an increasing trend among all treatments; the study conducted by Owheruo, Akpoghelie, Edo, Yousif, Jikah, Onyibe, Ugbune, Isoje, Igbuku, Ekokotu, Oghroro, Okpoghono, Essaghah and Agbo [37] on germinated sesame seeds showed enhanced protein content. The increase could be due to the hydrolysis of complex organic compounds by endogenous enzymes and enhanced proteolytic activity, which improves nutrient availability and reduces anti-nutrients [38]. During the imbibition phase, the hydrolysis of storage proteins is activated by proteases, yielding free amino acids and low-molecular-weight peptides in the germinating seed [22] Similarly, de novo synthesis directly contributes to an increased nitrogen content of the seed [39]. The protein content in this study was determined using the Kjeldahl method, which uses total nitrogen content and is multiplied by a known factor (6.25). This method overestimates protein content because the nitrogen content includes free amino acids, nucleic acids, and amides [40]. Further studies should use more robust quantification methods, such as the Bradford assay, to separate protein and non-protein fractions. Another study on germinated sesame seeds observed increased protein content due to the increased water activity because of hormonal or compositional changes. The soybeans also depicted an increase with longer germination duration [41].

The crude fat content in this current study showed a decreasing trend, with control S0 showing the highest fat content, inferring it to be a good source of fat. A study on germinated sesame seeds conducted by Maria and Victoria [41] showed a decreasing trend of fat content; the reduction in the fat content is due to the enhanced activity of lipase enzyme during germination, which helps in the breakdown of fat into fatty acids and glycerol molecules. Hahm, et al. [42] stated that reduced lipid content in germinated sesame seed is attributed to providing energy for protein synthesis during the seed growth. A similar trend of lipid reduction in soybeans has also been reported.

Crude fiber and carbohydrate content increased significantly in the current research; a longer germination period was directly proportional to the fiber content and NFE. The increased fiber content was in accordance with the results reported by Maria and Victoria [41] in germinated sesame seeds till 96 h. Buckwheat sprouts showed an increase in fiber content till 8th day of germination. Another research on sprouted buckwheat flour indicated improved fiber content; the increase was due to fibrous material such as cellulose, hemicellulose, and lignin [43]. Fiber content can be influenced by soaking time given to the seed before germination. As peanuts and soya beans were subjected to germination, the results were similar to this study, in comparison to the results of soaked peanuts, mung beans, wheat and barley, which decreased [44]. The increase in NFE with germination is largely explained by its formula as a difference, obtained by subtracting moisture, ash, crude protein, crude fat, and crude fiber from 100%. This aligns with the research on soluble sugar accumulation during germination, where soybean germination increased total sugars by 19% and total dietary fiber from 32% to 73%. This result, however, contrasts with previous sesame studies reporting a decline in NFE due to hydrolysis of stored carbohydrates into simple sugars utilized for embryo respiration. This divergence may reflect differences in germination temperature, duration, and drying conditions [41,45].

In the current study, total phenolic content (TPC) increased gradually; this trend is supported by a study evaluating phenolics in germinated and non-germinated wheat. It stated that longer germination was directly linked to the increase in TPC. This increase was due to the synthesis of phenolics released from the bound form, while extractable phenolics typically increased [46]. The findings of this study are comparable to the results for increased TPC in germinated black chickpea; the observed increase may be due to enzymatic degradation of the black chickpea structure, which may have helped enhance the phenolic content [47]. Black and white sesame seeds were compared for phenolic content; both of the seeds showed enhanced phenolic content upon germination, and the white germinated seeds showed thrice the amount of phenolic compounds than raw sesame seeds [41]. The findings of this current study were similar to the results of increased phenolic compounds in soybeans as well [48]. In conclusion, germination influences phenolic content by the activation of enzymes and biochemical pathways [41]. In this research, elongated germination reduced the flavonoid content. This can be supported by the study by Kim, et al. [49], which reported a decrease in seed coat flavonoids in two lentil cultivars during germination. It may be due to seed coat degradation during germination that reduces the flavonoid content of lentils. The findings of this research are in contrast with the improved flavonoid content in germinated moong grains; this increase in TFC may be because of the germination process that produces flavonoid compounds in the new seedling [50]. The study on germinated flaxseed showed an inverse relation with longer germination durations; flavonoids increased mid-germination and started declining in later stages, and the decrease can be justified as they might transform into bound forms, used during cell wall or lignin synthesis [51].

The individual flavonoids increased during mid-germination and decreased during the elongated germination stage. A study conducted by Thepthanee, et al. [52] analyzed changes in sunflower seeds by different processes; they also noted that elongated germination decreased the flavonoid content in striped sunflower seeds. An increase in rutin content was observed in germinated common bean (*Phaseolus vulgaris*), but it started declining at 96 h of germinated sample [53]; likewise, in the current study, it started declining in later stages of germination. The results for individual flavonoids are in line with the findings of TFC: they all peaked at S2, except for isorhamnetin, which peaked at S3 in later stages, and then declined in their respective contents with elongated germination. The increase is due to the activation of enzymes such as PAL, C4H, and 4CL. A study on germinated buckwheat and sorghum flour showed increased values of catechin and epicatechin after 48 h of germination, which later declined after 72 h. Similar results to this study, where they both increased at S2 and later decreased [54].

In the present research, the highest DPPH was observed in (S1), and the lowest was in the sesame seeds germinated for 120 h (S5). The findings of the study on germinated moong grains stated an increase in mid-germination and a decrease in the later stage of germination [49]. This finding can be used to support the DPPH trend. The increased antioxidant activity can be supported by the research of Mares, Passos and Menezes [35]. In the study mentioned, he relates high DPPH to enhanced synthesis and release of free phenolic compounds, due to activation of the metabolic pathway and enzymatic hydrolysis of bound phenolics.

The ABTS radical scavenging activity aligns with the analysis conducted by Zhi, et al. [55], which states that ABTS increased in early stages and peaked at mid-germination, and then declined in the later stages of germination. The present study aligns with the decrease in ABTS activity with longer germination, as ABTS activity began to decline after 96 h of germination in sesame seeds, reaching a minimum in S5 (sesame seeds germinated for 120 h). The increase in the early stages is most likely due to the release of phenolic compounds by enzymes, and the decline is attributable to the utilization of antioxidant compounds during germination [55]. The variable ABTS trend may be due to hydrolytic and polyphenol oxidase enzymes synthesized during germination [56].

Consistent with the findings in germinated triticale [57], in which it was reported the L values rose post germination due to the enzymatic pigment degradation, the present study revels similar results as the highest lightness was in early stages of germination in S1 (sesame seeds germinated for 24 h) followed by darkening in later stages as S3 (sesame seeds germinated for 72 h) to S5 (sesame seeds germinated for 120 h), as the color compounds are broken down. a* and b* values reduced during germination, which may be because of degradation of the color compounds such as carotenoids and chlorophylls [58]. The reduction in color values during germination may be due to carbohydrate and protein hydrolysis [47]. The constant increase in chroma until mid-germination noted in the study was closely aligned with the outcomes of Ramos-Pacheco, et al. [59], where enhanced saturation was observed at 72 h of germination in the quinoa sample. This saturation was due to the high concentration of carotenoid pigments. The decline in hue angle after S2 can be attributed to the enzymatic activity during germination [59].

## 5. Conclusions

The present study demonstrates that germination significantly influences the physicochemical properties and antioxidant profile of sesame seeds (*Sesamum indicum* L.), with effects strongly dependent on germination duration. Short-term germination enhances antioxidant activity and phytochemical content, whereas prolonged germination reduces antioxidant capacity. Although higher polyphenol levels were observed at extended germination periods (96–120 h), these benefits were offset by reduced antioxidant efficiency and visible mold growth, compromising product safety and quality. A negative correlation between germination time and antioxidant activity further highlights the need for optimization. Notably, sesame seeds germinated for 48 h exhibited a balanced profile, with relatively higher flavonoid content and satisfactory antioxidant activity, making this duration conducive for functional applications. Therefore, controlled germination, particularly around 48 h, is recommended to maximize the nutritional and functional potential of sesame seeds while ensuring safety and quality for their incorporation into value-added food products.

### Limitations and Recommendations

Phytochemical quantification relied exclusively on UV-visible spectrophotometry, which lacks the selectivity required for compound identification; chromatographic confirmation using HPLC or LC-MS is recommended in future work. Germination was carried out under ambient conditions (25 ± 2 °C) without formal environmental control for relative humidity (30–35%), which may otherwise have affected the outcomes. Furthermore, the seeds were procured from the local market with no information on cultivar purity or geographic origin, introducing potential sources of biological variability. Finally, microbiological assessment was not conducted, which can be monitored in future studies. In particular, the mycotoxin content can be determined if any mold growth is detected. These gaps can be prioritized in subsequent studies so that a more precise process may be devised for germination and microgreen production. Furthermore, enzymatic activity analyses will further help in understanding the metabolic pathways of germination-induced modifications in the nutritional profile of seeds.

## Figures and Tables

**Table 1:** Treatment plan.

Treatment	Description
S0	Dry sesame seeds
S1	Sesame seeds germinated for 24 h after soaking for 5 h
S2	Sesame seeds germinated for 48 h after soaking for 5 h
S3	Sesame seeds germinated for 72 h after soaking for 5 h
S4	Sesame seeds germinated for 96 h after soaking for 5 h
S5	Sesame seeds germinated for 120 h after soaking for 5 h

**Table 2:** Individual flavonoids with their respective extinction coefficient and molecular weight.

Flavonoids	λ Max (nm)	Extinction Coefficient (L·mol^−1^cm^−1^)	Molecular Weight (g/mol)	Reference
Rutin	262	1.6429 × 10^6^	610.5	[27]
Epicatechins	262	3.950 × 10^3^	290.27	[29,30]
Luteolin	280	3.7646 × 10^4^	286.24	[31]
Isorhamnetin	280	8.958 × 10^3^	316.26	[27]
β-Catechins	295	3.950 × 10^3^	290.27	[32]
Procyanidins	321	8.050 × 10^3^	594.5	[32]
Kaempferol	365	2.1242 × 10^4^	286.24	[27]
Quercetin	415	6.3197 × 10^2^	302.23	[27]

The final results were reported as mg/100 g of sesame seeds.

**Table 3:** Proximate composition (%) of germinated and non-germinated sesame seeds.

Treatments	Moisture (%)	Ash (%)	Crude Protein (%)	Crude Fat (%)	Crude Fiber (%)	Nitrogen-Free Extract (%)
S0	4.39 ± 0.09 ^c^	1.98 ± 0.02 ^ab^	18.64 ± 0.37 ^c^	45.33 ± 1.76 ^a^	5.91 ± 0.12 ^c^	23.75 ± 1.59 ^b^
S1	6.59 ± 0.22 ^b^	2.12 ± 0.22 ^a^	19.57 ± 0.65 ^c^	37.33 ± 1.26 ^b^	10.90 ± 0.36 ^a^	23.49 ± 1.52 ^b^
S2	9.88 ± 0.22 ^a^	1.74 ± 0.19 ^a–c^	20.99 ± 0.46 ^b^	30.50 ± 1.00 ^c^	12.38 ± 0.27 ^a^	24.52 ± 1.88 ^b^
S3	10.05 ± 0.15 ^a^	1.32 ± 0.30 ^c^	21.81 ± 0.33 ^ab^	26.67 ± 1.61 ^d^	12.63 ± 0.19 ^a^	27.53 ± 2.42 ^b^
S4	9.68 ± 0.16 ^a^	1.81 ± 0.29 ^a–c^	22.00 ± 0.37 ^ab^	22.33 ± 1.53 ^e^	11.49 ± 0.20 ^b^	32.69 ± 1.13 ^a^
S5	9.63 ± 0.24 ^a^	1.49 ± 0.01 ^bc^	22.55 ± 0.56 ^a^	19.17 ± 1.04 ^e^	12.53 ± 0.31 ^a^	34.64 ± 2.01 ^a^

Results are the mean ± standard deviation of triplicates. Significant differences among treatments are represented by different letters in the same column.

**Table 4:** Means for phytochemicals in germinated and non-germinated sesame seeds.

Treatments	Total Polyphenol Content (mg GAE/100 g DW)	Total Flavonoid Content (mg QE/100 g DW)
S0	29.93 ± 0.73 ^d^	46.66 ± 3.37 ^b^
S1	33.99 ± 3.32 ^d^	28.50 ± 5.71 ^c^
S2	26.18 ± 3.60 ^d^	53.15 ± 3.37 ^a^
S3	58.42 ± 1.97 ^c^	37.08 ± 0.78 ^c^
S4	90.42 ± 6.61 ^b^	21.04 ± 3.29 ^d^
S5	137.28 ± 6.01 ^a^	11.94 ± 3.91 ^e^

Results are the mean ± standard deviation of triplicates. Significant differences among treatments are represented by different letters in the same column.

**Table 5:** Means for flavonoid (mg/100 g DW) profile of germinated and non-germinated sesame seed.

Treatments	Rutin	Isorhamnetin	Luteolin	Kaempferol	Quercetin	Procyanidin	β-Catechin	Epicatechin
S0	0.070 ± 0.006 ^c^	6.95 ± 0.52 ^b^	1.50 ± 0.11 ^c^	0.96 ± 0.05 ^c^	94.2 ± 7.12 ^c^	13.89 ± 0.51 ^c^	13.19 ± 0.59 ^c^	13.87 ± 1.19 ^c^
S1	0.058 ± 0.004 ^cd^	6.23 ± 0.57 ^b^	1.34 ± 0.13 ^c^	0.66 ± 0.01 ^c^	84.45 ± 7.77 ^c^	13.41 ± 0.62 ^c^	12.42 ± 0.94 ^c^	11.55 ± 0.64 ^c^
S2	0.110 ± 0.008 ^a^	7.40 ± 0.39 ^b^	2.23 ± 0.08 ^a^	1.43 ± 0.14 ^a^	140.04 ± 5.24 ^a^	21.64 ± 0.70 ^a^	19.97 ± 1.65 ^a^	21.74 ± 1.63 ^a^
S3	0.094 ± 0.002 ^b^	8.91 ± 0.32 ^a^	1.92 ± 0.07 ^b^	1.20 ± 0.06 ^b^	120.74 ± 4.3 ^b^	19.14 ± 0.78 ^b^	17.31 ± 0.83 ^b^	18.42 ± 0.48 ^b^
S4	0.049 ± 0.002 ^d^	4.30 ± 0.27 ^c^	0.93 ± 0.06 ^d^	0.66 ± 0.05 ^d^	58.21 ± 3.57 ^d^	9.64 ± 0.48 ^d^	8.01 ± 0.43 ^d^	1.64 ± 0.04 ^d^
S5	0.056 ± 0.002 ^d^	4.93 ± 0.46 ^c^	1.06 ± 0.10 ^d^	0.58 ± 0.02 ^d^	66.78 ± 6.20 ^d^	3.11 ± 0.10 ^e^	9.07 ± 0.45 ^d^	1.13 ± 0.02 ^d^

Results are the mean ± standard deviation of triplicates. Significant differences among treatments are represented by different letters in the same column.

**Table 6:** Means for antioxidant profile of non-germinated and germinated sesame seeds.

Treatments	DPPH (%)	ABTS (%)
S0	78.58 ± 0.16 ^c^	80.68 ± 1.81 ^a^
S1	91.36 ± 0.24 ^a^	54.89 ± 0.99 ^d^
S2	82.07 ± 1.01 ^b^	76.10 ± 1.90 ^b^
S3	77.78 ± 1.29 ^c^	66.51 ± 1.80 ^c^
S4	30.22 ± 0.74 ^d^	79.91 ± 0.16 ^ab^
S5	11.26 ± 0.29 ^e^	58.74 ± 2.15 ^d^

Results are the mean ± standard deviation of triplicates. Significant differences among treatments are represented by different letters in the same column.

**Table 7:** Means for color analysis of non-germinated and germinated sesame seeds.

Treatments	L	a*	b*	Chroma	Hue
S0	68.70 ± 0.10 ^ab^	3.90 ± 0.40 ^a^	21.47 ± 0.67 ^bc^	50.73 ± 0.81 ^bc^	5.55 ± 0.71 ^c^
S1	69.77 ± 1.17 ^a^	4.35 ± 0.15 ^a^	23.10 ± 1.45 ^ab^	54.90 ± 2.75 ^ab^	5.32 ± 0.45 ^c^
S2	65.77 ± 1.18 ^ab^	2.20 ± 0.20 ^b^	26.20 ± 0.35 ^a^	56.80 ± 0.80 ^a^	11.98 ± 1.12 ^a^
S3	64.57 ± 2.83 ^b^	1.97 ± 0.15 ^b^	22.37 ± 0.78 ^bc^	48.67 ± 1.86 ^cd^	11.40 ± 0.50 ^ab^
S4	59.57 ± 4.16 ^bc^	2.05 ± 0.05 ^b^	21.93 ± 1.77 ^bc^	47.97 ± 3.54 ^cd^	10.70 ± 0.88 ^ab^
S5	59.95 ± 1.63 ^c^	2.30 ± 0.40 ^b^	19.37 ± 1.14 ^c^	43.33 ± 1.51 ^d^	8.65 ± 1.99 ^b^

Results are the mean ± standard deviation of triplicates. Significant differences among treatments are represented by different letters in the same column.

## Data Availability

The original contributions presented in this study are included in the article and Supplementary Materials. Further inquiries can be directed to the corresponding authors.

## Supplementary Materials

The following supporting information can be downloaded at: https://www.mdpi.com/article/10.3390/biotech15030060/s1, Table S1: Absorbances for Polyphenols and Flavonoids at Their Respective Wavelengths; Table S2: Absorbances for Antioxidant Assays at Their Respective Wavelengths; Table S3: Values for Color Parameters.

## Abbreviations

The following abbreviations are used in this manuscript:

QE	Quercitrin Equivalent
FAO	Food and Agriculture Organization
PPM	Parts Per Million
NFE	Nitrogen-Free Extract
DPPH	2,2-diphenyl-1-picrylhydrazyl
ABTS	2,2′-azino-bis (3-ethylbenzothiazoline-6-sulfonic acid)
GAE	Gallic Acid Equivalent
