# Supplementary material for: Germination-Induced Variations in Proximate Composition, Phytochemical Profile and Antioxidant Activities of White Sesame Seeds: A Green Approach for Nutritional Modification
Source: BioTech (Basel). 2026 Jul 29;15(3):60. doi: 10.3390/biotech15030060 (PMC13510433; doi:10.3390/biotech15030060)
Supplement: Supplementary file 1 [file biotech-15-00060-s001.zip › biotech-4331664-supplementary.pdf]

Supplementary Materials: Germination Induced Variations in Proximate Composition, Phytochemical Profile and Antioxidant Activities of White Sesame Seeds: A Green Approach for Nutritional Modification

Oneeza Anwar, Iahtisham-Ul-Haq, Ramiz Arif, Waqas Ahmed and Hafiz Ansar Rasul Suleria

Table S1. Absorbances for Polyphenols and Flavonoids at Their Respective Wavelengths.

Codes	Replica-tion	TPC	TFC	Rutin	Isorham-netin	Luteolin	Kaempfer ol	Quercetin	Procya-nidins	Epicate-chins	β-Cate-chins
S0	1	0.307	0.477	2.041	2.161	2.161	0.809	0.694	2.235	2.041	2.070
	2	0.319	0.477	2.387	2.499	2.499	0.901	0.750	2.320	2.387	2.129
	3	0.303	0.533	2.365	2.431	2.431	0.855	0.988	2.405	2.365	2.260
S1	1	0.371	0.362	1.782	1.911	1.911	0.597	0.426	2.239	1.782	1.872
	2	0.313	0.343	1.989	2.148	2.148	0.587	0.398	2.135	1.989	2.034
	3	0.384	0.353	1.886	2.298	2.298	0.592	0.496	2.343	1.886	2.179
S2	1	0.308	0.572	3.845	2.662	3.662	1.387	1.159	3.747	3.845	3.569
	2	0.267	0.581	3.470	2.470	3.470	1.270	1.217	3.534	3.470	3.079
	3	0.226	0.521	3.333	2.410	3.410	1.153	0.945	3.558	3.333	3.133
S3	1	0.612	0.395	3.047	2.963	2.963	1.043	0.573	3.056	2.929	2.718
	2	0.635	0.408	3.008	2.972	2.972	1.029	0.596	3.221	3.008	2.784
	3	0.657	0.408	3.086	3.154	3.154	1.135	0.641	3.312	3.086	2.977
S4	1	0.913	0.274	1.660	1.550	1.550	0.629	0.503	1.688	0.274	1.378
	2	1.052	0.261	1.503	1.371	1.371	0.551	0.402	1.530	0.261	1.239
	3	1.033	0.268	1.582	1.461	1.461	0.590	0.333	1.609	0.268	1.309
S5	1	1.602	0.186	1.803	1.814	1.814	0.534	0.380	0.534	0.186	1.554
	2	1.534	0.182	1.870	1.706	1.706	0.503	0.315	0.503	0.182	1.409
	3	1.465	0.184	1.735	1.507	1.507	0.519	0.234	0.519	0.184	1.482

Table S2. Absorbances for Antioxidant Assays at Their Respective Wavelengths.

Codes	Replication	DPPH	ABTS
S0	1	0.294	0.165
	2	0.295	0.153
	3	0.292	0.184
S1	1	0.122	0.400
	2	0.116	0.383
	3	0.117	0.389
S2	1	0.261	0.226
	2	0.234	0.197
	3	0.242	0.198
S3	1	0.305	0.298
	2	0.317	0.272
	3	0.292	0.300
S4	1	0.945	0.170
	2	0.958	0.175
	3	0.965	0.173
S5	1	1.212	0.370
	2	1.220	0.336
	3	1.215	0.366

Table S3. Values for Color Parameters.

Treatment	L	a*	b*	Chroma	Hue
S0	68.7	3.9	21.9	51.6	5.62
S0	68.8	4.3	20.7	50	4.81
S0	68.6	3.5	21.8	50.6	6.23
S1	70.8	4.35	24.5	57.7	5.63
S1	68.5	4.2	23.2	54.8	5.52
S1	70	4.5	21.6	52.2	4.8
S2	66.5	2	26.4	56.8	13.2
S2	64.4	2.2	25.8	56	11.73
S2	66.4	2.4	26.4	57.6	11
S3	64.6	2	22.6	49.2	11.3
S3	61.3	1.8	21.5	46.6	11.94
S3	66.1	2.1	23	50.2	10.95
S4	66.3	2.1	23.1	50.4	11
S4	60.9	2.05	19.9	43 .9	9.71
S4	62.9	2	22.8	49.6	11.4
S5	54.9	2.3	19.7	44	8.57
S5	58.8	1.9	20.3	44.4	10.68
S5	61.1	2.7	18.1	41.6	6.7
